# Preoperative Thrombocytopenia Increases In-Hospital Burden but Not Postoperative Adverse Events After Total Knee Arthroplasty

**DOI:** 10.3390/jcm15166187

**Published:** 2026-08-10

**Authors:** Jisu Park, Moon Jong Chang, Minji Han, Jung-Wee Park, Tae Woo Kim, Young-Kyun Lee, Chong Bum Chang, Seung-Baik Kang

**Affiliations:** 1 Department of Orthopedic Surgery, Seoul National University College of Medicine, SMG-SNU Boramae Medical Center, Seoul 07061, Republic of Korea; pjswltn@gmail.com (J.P.); moonjongchang@gmail.com (M.J.C.); 2 Big Data Center, Seoul National University Bundang Hospital, Seongnam 13620, Republic of Korea; mj830@snu.ac.kr; 3 Department of Orthopedic Surgery, Seoul National University College of Medicine, Seoul National University Bundang Hospital, Seongnam 13620, Republic of Korea; jwepark@gmail.com (J.-W.P.); ykleemd@gmail.com (Y.-K.L.); ccbknee@gmail.com (C.B.C.); 4 Knee Center, Gangnam St. Peter’s General Hospital, Seoul 07061, Republic of Korea; ossbkang@gmail.com

**Keywords:** total knee arthroplasty, thrombocytopenia, perioperative burden, adverse events

## Abstract

***Background/Objectives***: Thrombocytopenia increases bleeding tendency and may complicate perioperative management in total knee arthroplasty (TKA), yet data on both in-hospital burden and post-discharge adverse events are limited. This study evaluated the association between preoperative platelet count and outcomes after primary TKA. ***Methods***: In total, 3474 primary TKAs performed for osteoarthritis at a single institution (2008–2023) were retrospectively reviewed. Patients were classified as Normal, Mild, Moderate, or Severe by preoperative platelet count; the Severe group (*N* = 6) was excluded from comparative analyses and reported descriptively. Inverse probability of treatment weighting (IPTW) balanced baseline covariates. In-hospital outcomes were red blood cell (RBC) transfusion, intensive care unit (ICU) admission, and length of stay; follow-up outcomes were 30-day readmission, 1-year periprosthetic joint infection (PJI), and 1-year mortality. ***Results***: After IPTW, the RBC transfusion rate rose with lower platelet count (Normal, 14.0%; Mild, 27.0%; Moderate, 32.5%; *p* < 0.001), and transfusion volume peaked in the Moderate group. ICU admission was more frequent (Moderate, 6.0%; Normal, 0.7%; Mild, 0.4%; *p* = 0.002), and length of stay increased with severity (*p* = 0.008). Readmission, 1-year PJI, and mortality were comparable across groups. In multivariable analysis, moderate thrombocytopenia was independently associated with RBC transfusion (adjusted odds ratio [aOR], 3.26; 95% confidence interval [CI], 1.51–6.59; *p* < 0.001) and ICU admission (aOR, 5.46; 95% CI, 1.37–17.41; *p* = 0.008), but not PJI or mortality. ***Conclusions***: Preoperative thrombocytopenia—particularly in the mild-to-moderate range evaluated in this study—increased perioperative burden after TKA, with higher RBC transfusion and ICU utilization, but was not associated with a significant increase in major postoperative adverse events during 1-year follow-up. Even if not causally responsible, a low preoperative platelet count is a routinely available marker that may flag patients requiring closer perioperative attention. As the associated burden appears largely limited to the hospitalization period, such attentive in-hospital management may allow these patients to achieve favorable outcomes.

## 1. Introduction

Thrombocytopenia is a hematologic condition characterized by a reduced platelet (PLT) count and is traditionally associated with an increased risk of bleeding [1]. Compared with a normal PLT count (≥150,000/µL), it can be classified into mild (100,000–150,000/µL), moderate (50,000–100,000/µL), and severe (<50,000/µL) [2]. Given the critical role of PLT in hemostasis, preoperative thrombocytopenia has the potential to influence perioperative bleeding risk, transfusion requirement, and postoperative recovery [3,4].

Total knee arthroplasty (TKA) is an effective and widely performed procedure for end-stage knee osteoarthritis [5,6]. With the adoption of the Enhanced Recovery After Surgery (ERAS) protocols, contemporary perioperative management emphasizes early mobilization, reduced length of hospital stay, and rapid functional recovery [7,8]. However, postoperative factors such as anemia and the need for blood transfusion can impede these goals and delay discharge [9]. In this context, patients with preoperative thrombocytopenia may be at risk for increased perioperative burden, prolonged hospitalization, and potentially worse outcomes after discharge.

Despite these concerns, the impact of thrombocytopenia on outcomes after TKA remains incompletely understood. Prior studies have reported conflicting results regarding transfusion rates, length of hospital stay, and postoperative complications in patients with thrombocytopenia undergoing total joint arthroplasty [10,11,12,13]. Moreover, many existing studies are based on small sample sizes or rely on large administrative databases with limited perioperative detail, making it difficult to assess both perioperative burden and post-discharge adverse events in a comprehensive manner [10,11,14,15,16,17]. Importantly, few studies have simultaneously evaluated perioperative burden and post-discharge adverse events.

Therefore, the purpose of this study was to evaluate the association between preoperative PLT count and both in-hospital outcomes and postoperative adverse events during follow-up after primary TKA. It was hypothesized that lower preoperative PLT count would be associated with increased perioperative burden but would not necessarily translate into higher rates of major postoperative adverse events during follow-up.

## 2. Materials and Methods

### 2.1. Study Design and Patient Selection

This retrospective study included patients who underwent primary TKA for osteoarthritis at a single institution between 2008 and 2023. Because the institution’s electronic medical record (EMR) system was updated during the study period, only patients with complete data were considered eligible, which yielded 3474 TKA cases for analysis. When a patient underwent bilateral TKA during a single admission period, both procedures were analyzed as a single case, and bilaterality was incorporated as a covariate. All baseline variables presented in Table 1 were complete for the included cases; cases with incomplete records following the EMR transition were not eligible for inclusion, and no imputation was performed.

Patients were stratified into four groups according to their PLT counts: the Normal group (PLT count ≥ 150,000/µL), the Mild group (100,000/µL ≤ PLT count < 150,000/µL), the Moderate group (50,000/µL ≤ PLT count < 100,000/µL), and the Severe group (PLT count < 50,000/µL). Accordingly, the Normal, Mild, Moderate, and Severe groups comprised 3206, 201, 61, and 6 cases. Given the very small number of patients in the Severe group, this group was excluded from the primary comparative analyses. The primary study population, therefore, consisted of the Normal, Mild, and Moderate groups, in which the inverse probability of treatment weighting (IPTW) method was applied to adjust for differences in group size and covariate distribution. Age, sex, height, use of tranexamic acid (TXA), use of closed suction drainage, bilaterality, and the anesthesia method were used for the IPTW. After the IPTW, the baseline characteristics were comparable among the three groups except for the baseline PLT and hemoglobin levels (Table 1).

Patients with severe thrombocytopenia were retained in the overall cohort and were analyzed descriptively without statistical adjustment or between-group comparison; their clinical outcomes were reported in Supplementary Tables S5 and S6. This study was approved by the institutional review board (IRB) of the author’s institution (IRB number 30-2024-18).

### 2.2. Medical Records Review

The medical records of the included cases were retrospectively reviewed. Patients’ age, sex, height, and weight were collected. Also, past medical history was reviewed, and the modified Charlson Comorbidity Index (CCI) score was calculated. While reviewing the medical history, the use of antiplatelet or anticoagulant agents was also checked.

A total of three surgeons performed the surgery. All three surgeons used the medial parapatellar approach and targeted mechanical alignment. The surgery was performed under spinal or general anesthesia based on the patients’ medical condition. The use of TXA was recorded. In most cases, a closed suction drain was used. The operation time and estimated blood loss (EBL) during surgery were reviewed.

During the perioperative period, if the hemoglobin level fell below 7 g/dL or if there were symptoms or signs of dizziness, chest pain, rapid heart rate, or low blood pressure, red blood cells (RBCs) were transfused. The same institutional transfusion threshold was applied throughout the study period, although transfusion practice in the earlier years of the cohort was more liberal than in recent years, reflecting a broader shift toward restrictive transfusion strategies. Whether patients received RBC transfusion and the amount of transfusion were reviewed. The use of the intensive care unit (ICU) and the length of hospital stay were reviewed. In this study, ICU admission was defined as direct transfer from the operating room to the ICU immediately after surgery, without passing through the post-anesthesia care unit, rather than ICU admission at any later point during hospitalization. This decision was made intraoperatively at the discretion of the attending anesthesiologist and surgeon, and was indicated for planned close monitoring in patients with significant medical comorbidities or anesthetic risk, for intraoperative bleeding or hemodynamic instability, or for intraoperative cardiopulmonary events. Oozing that persisted for more than 3 days after surgery was defined as prolonged oozing, and whether it occurred during the hospitalization period was checked. During follow-up periods, 30-day readmission, periprosthetic joint infection (PJI) within the first postoperative year, and 1-year mortality were reviewed.

The management of thrombocytopenia itself was individualized. Preoperatively, patients with thrombocytopenia—particularly those with a moderate or severe reduction or an unclear etiology—were referred for hematology consultation and evaluation of the underlying cause, and the decision to proceed with surgery was made on a case-by-case basis rather than based on a fixed platelet threshold or routine prophylactic correction.

### 2.3. Statistical Analysis

All statistical analyses were conducted using R (version 4.3.3; R Foundation for Statistical Computing, Vienna, Austria). To address potential confounding and imbalance in group sizes, IPTW based on generalized propensity scores was applied. Propensity scores were estimated using a multinomial logistic regression model, with the preoperative PLT group as the dependent variable and baseline covariates as predictors. Standardized mean differences (SMDs) were used to assess balance across groups.

For outcome comparisons, a survey-weighted design object was constructed using the *svydesign* function in the *survey* package, with IPTWs applied as sampling weights. Continuous variables were analyzed using IPTW-adjusted linear regression models by *svyglm*, and group-wise weighted means with 95% confidence intervals were obtained using the *svyby* function. Categorical variables were analyzed using *svyglm* with a quasibinomial family and logit link function, and overall group effects were evaluated using Wald tests implemented through *regTermTest*.

In addition to the IPTW-adjusted analyses, conventional multivariable logistic regression was performed to identify independent factors associated with RBC transfusion, ICU admission, 1-year PJI, and 1-year mortality. Because baseline hemoglobin is closely correlated with PLT count and could not be completely balanced by IPTW, this model served as a confirmatory analysis that directly adjusted for baseline hemoglobin together with other covariates. Variables with *p* < 0.10 in univariable analysis, or those considered clinically relevant including baseline hemoglobin, were included in the multivariable model, and odds ratios (ORs) with 95% confidence intervals (CIs) were calculated.

A sensitivity analysis was conducted by combining patients with severe thrombocytopenia with the Moderate group to assess the robustness of the primary findings. All statistical tests were two-sided, and *p*-values < 0.05 were considered statistically significant.

## 3. Results

### 3.1. Perioperative Burden According to Preoperative PLT Count

Perioperative outcomes differed by preoperative PLT group (Table 2). Although EBL was comparable among the three groups, the incidence of RBC transfusion increased in the groups with lower PLT counts, occurring in 14.0% of the Normal group, 27.0% of the Mild group, and 32.5% of the Moderate group (*p* < 0.001). The transfused RBC amount also differed among the groups, with the Moderate group receiving the highest transfusion volume. Postoperative ICU admission was more frequent in the Moderate group (6.0%) compared with the Normal (0.7%) and Mild (0.4%) groups (*p* = 0.002). In addition, the length of hospital stay was longer with increasing severity of thrombocytopenia (*p* = 0.008). In contrast, the incidence of prolonged oozing did not differ among the three groups. Although operation time differed statistically among the groups, the absolute difference was small (< 4 min) and unlikely to be clinically meaningful.

### 3.2. Postoperative Adverse Events During Follow-Up

During the follow-up period, there were no meaningful clinical differences among the three groups. Readmission rates were low across all groups, and no clinically meaningful differences were observed. Also, 1-year PJI and 1-year mortality did not differ among the three groups (Table 2).

### 3.3. Multivariable Logistic Regression

Moderate thrombocytopenia was associated with an increased risk of perioperative RBC transfusion and ICU admission (Table 3). In a multivariable logistic regression analysis, patients with moderate thrombocytopenia had a significantly higher likelihood of RBC transfusion (adjusted odds ratio [aOR], 3.26; 95% CI, 1.51–6.59; *p* < 0.001) and ICU admission (aOR, 5.46; 95% CI, 1.37–17.41; *p* = 0.008) compared with those with normal PLT count. No significant results were observed regarding 1-year PJI (aOR, 3.14; 95% CI, 0.75–8.92; *p* = 0.061) and 1-year mortality (aOR, 3.06; 95% CI, 0.15–22.11; *p* = 0.343). In contrast, mild thrombocytopenia was not independently associated with RBC transfusion (aOR, 1.48; 95% CI, 0.91–2.39; *p* = 0.108), ICU admission (aOR, 0.52; 95% CI, 0.03–2.66; *p* = 0.535), 1-year PJI (aOR, 0.95; 95% CI, 0.23–2.60; *p* = 0.925), and 1-year mortality (aOR, 1.54; 95% CI, 0.08–9.98; *p* = 0.703). The results for all variables used in the multivariable logistic regression are presented in Supplementary Tables S1–S4.

### 3.4. Sensitivity and Descriptive Analyses

Sensitivity analysis that included patients with severe thrombocytopenia in the Moderate group demonstrated results consistent with those of the primary analysis (Supplementary Table S5). Outcomes in patients with severe thrombocytopenia were summarized descriptively and are presented in Supplementary Table S6.

## 4. Discussion

In this IPTW-adjusted retrospective study, the association between preoperative PLT count and perioperative outcomes in patients undergoing primary TKA was evaluated. Decreasing PLT count was associated with greater perioperative burden, whereas short- and mid-term major postoperative adverse events were comparable across PLT count groups. Multivariable analysis further identified moderate thrombocytopenia as an independent risk factor for perioperative RBC transfusion and ICU admission, but not PJI and mortality.

The first key finding of this study is that lower preoperative PLT count, particularly in the moderate thrombocytopenia range, was associated with increased in-hospital burden following TKA. This association was observed despite comparable operative characteristics and persisted after adjustment of baseline imbalances, indicating that moderate thrombocytopenia confers vulnerability during the immediate perioperative period. Consistent with this observation, multivariable analysis further supported moderate thrombocytopenia as an independent risk factor for RBC transfusion and ICU admission, reinforcing its clinical relevance in in-hospital management. These results are also consistent with previous studies. Go et al. compared patients undergoing hip arthroplasty with severe preoperative thrombocytopenia to those in the normal group and found higher transfusion rates and volumes in the thrombocytopenic group [13]. Another retrospective study by Bujnowski et al. demonstrated increased risk of blood transfusion in TKA patients with moderate thrombocytopenia [12]. The results regarding the length of hospital stay vary across the literature. Unlike the studies by Go and Bujnowski, which found no difference in hospital stay length, Malpani et al. and Wang et al. observed longer hospital stays in patients with thrombocytopenia [11,17]. In the current study, a longer hospital stay was observed in the thrombocytopenia groups. Of note, baseline hemoglobin remained lower in the thrombocytopenic groups even after IPTW. Because hemoglobin is strongly correlated with PLT count, it could not be completely balanced by weighting, and as a major determinant of transfusion, it may have contributed to the higher transfusion requirement observed in these groups. For this reason, multivariable logistic regression that directly adjusted for baseline hemoglobin was additionally performed, and moderate thrombocytopenia remained independently associated with RBC transfusion; nonetheless, the influence of residual hemoglobin imbalance should be considered when interpreting this association.

Unlike the in-hospital results, the occurrence of adverse events during the follow-up period was not associated with thrombocytopenia. Rates of major postoperative adverse events, including 30-day readmission, 1-year PJI, and 1-year mortality, remained low and were comparable across PLT count groups. These findings suggest that while moderate thrombocytopenia influences short-term perioperative management, it does not appear to compromise short- or mid-term postoperative safety after discharge. Previous studies have also demonstrated no increased readmission rate in patients with thrombocytopenia [10,12]. The results differ with respect to major adverse events such as PJI and mortality. Malpani et al. used a nationwide dataset and demonstrated increased risk of major adverse events in both abnormally low and high PLT patients [17]. However, only an analysis of the overall major adverse events was presented, and individual odds ratios for mortality and PJI were not provided. Also, their outcomes were confined to the 30-day postoperative follow-up. In another nationwide database study by Wang et al., in-hospital mortality and surgical complications increased in patients with thrombocytopenia, but they did not offer follow-up outcomes after discharge [11]. These database studies involve large cohorts, but a drawback is that they used code-based classification, so they did not provide details and follow-up data. In the 1-year follow-up results in this study, the risk of PJI and mortality was not associated with thrombocytopenia. From a clinical standpoint, these findings suggest that moderate thrombocytopenia should prompt optimization of perioperative blood management—including correction of preoperative anemia, routine TXA administration, and preparedness for transfusion—rather than serve as a reason to defer or preclude elective TKA.

This study has several limitations. First, its retrospective design inherently carries the risk of residual confounding despite the use of IPTW. Second, patients with severe thrombocytopenia were too few for meaningful statistical comparison and were therefore excluded from the comparative analyses and assessed descriptively only; accordingly, the present findings apply primarily to mild and moderate thrombocytopenia. Third, transfusion practices were based on institutional protocols and clinical judgment rather than a strictly standardized algorithm, and the 15-year study period encompassed evolving perioperative practice, including the wider adoption of ERAS protocols, a shift toward more restrictive transfusion strategies, and the routine use of TXA. Notably, TXA use, drain use, and anesthesia method were included as covariates in the IPTW model and were well balanced after weighting (Table 1). Because such temporal changes would be expected to affect all PLT groups non-differentially, they are unlikely to account for the observed between-group differences, although a residual influence cannot be excluded. Finally, this was a single-center study with a relatively small number of patients with moderate thrombocytopenia and low event rates for PJI and mortality. As a result, the confidence intervals for ICU admission and 1-year mortality were wide, and the study may have been underpowered to detect clinically meaningful differences; these estimates should be interpreted as indicating the direction rather than the precise magnitude of the associated risk, and the absence of a statistically significant association does not exclude a true effect. These negative findings should be regarded as hypothesis-generating rather than conclusive. As follow-up was limited to one year, the impact of thrombocytopenia on long-term implant survival or late complications could not be determined.

## 5. Conclusions

Preoperative thrombocytopenia—particularly in the mild-to-moderate range evaluated in this study—increased perioperative burden after TKA, with higher RBC transfusion and ICU utilization, but was not associated with a significant increase in major postoperative adverse events during 1-year follow-up. Even if not causally responsible, a low preoperative platelet count is a routinely available marker that may flag patients requiring closer perioperative attention. As the associated burden appears largely limited to the hospitalization period, such attentive in-hospital management may allow these patients to achieve favorable outcomes.

## Figures and Tables

**Table 1:** Demographic data of the primary study population before and after IPTW adjustment.

	Unweighted			SMD	Weighted			SMD
	Normal	Mild	Moderate		Normal	Mild	Moderate	
Number	3206	201	61		3468.7	3394.6	3459.7	
Age (year)	71.9 ± 6.3	73.3 ± 6.2	73.6 ± 6.4	0.181	72.0 ± 6.4	72.4 ± 6.4	72.5 ± 6.0	0.059
Sex				0.226				0.040
Male	444 (13.8%)	55 (27.4%)	9 (14.8%)		509.6 (14.7%)	566.2 (16.7%)	501.6 (14.5%)	
Female	2762 (86.2%)	146 (72.6%)	52 (85.2%)		2959.1 (85.3%)	2828.4 (83.3%)	2958.1 (85.5%)	
Height (cm)	153.0 ± 7.2	155.0 ± 7.9	153.3 ± 8.1	0.175	153.1 ± 7.3	153.4 ± 7.2	153.6 ± 7.5	0.040
Weight (kg)	63.4 ± 10.0	64.4 ± 10.6	63.9 ± 11.6	0.063	63.5 ± 10.0	63.4 ± 10.4	64.7 ± 11.7	0.082
Baseline lab								
PLT (×10^3^/µL)	240.5 ± 52.6	132.1 ± 12.4	82.0 ± 14.3	3.567	240.0 ± 52.5	132.9 ± 12.2	81.4 ± 14.4	3.595
Hb (g/dL)	12.7 ± 1.4	11.5 ± 2.2	11.3 ± 2.1	0.526	12.7 ± 1.4	11.7 ± 2.1	11.6 ± 1.7	0.468
CCI score	2.7 ± 0.7	2.9 ± 0.7	3.0 ± 0.7	0.208	2.7 ± 0.7	2.7 ± 0.7	2.8 ± 0.7	0.094
AntiPLT/Anticoagulant	897 (28.0%)	60 (29.9%)	21 (34.4%)	0.093	982.0 (28.3%)	958.2 (28.2%)	1161.6 (33.6%)	0.077
TXA use	2582 (80.5%)	161 (80.1%)	38 (62.3%)	0.275	2782.3 (80.2%)	2740.5 (80.7%)	2797.9 (80.9%)	0.011
Drain use	2962 (92.4%)	193 (96.0%)	58 (95.1%)	0.104	3213.8 (92.7%)	3113.6 (91.7%)	3306.0 (95.6%)	0.105
Bilaterality				0.311				0.051
Unilateral	1657 (51.7%)	135 (67.2%)	45 (73.8%)		1837.9 (53.0%)	1830.2 (53.9%)	1731.8 (50.1%)	
Bilateral	1549 (48.3%)	66 (32.8%)	16 (26.2%)		1630.8 (47.0%)	1564.4 (46.1%)	1727.9 (49.9%)	
Anesthesia				0.208				0.051
Spinal	2146 (66.9%)	104 (51.7%)	38 (62.3%)		2288.1 (66.0%)	2229.1 (65.6%)	2156.4 (62.3%)	
General	1060 (33.1%)	97 (48.3%)	23 (37.7%)		1180.6 (34.0%)	1165.5 (34.3%)	1303.3 (37.7%)	

Values are presented as mean ± standard deviation or number (%), unless otherwise indicated. An SMD < 0.1 was considered indicative of adequate covariate balance. IPTW: inverse probability of treatment weighting, SMD: standardized mean difference, PLT: platelet count, Hb: hemoglobin, CCI: Charlson Comorbidity Index, TXA: tranexamic acid.

**Table 2:** Clinical outcomes of the primary study population (IPTW adjusted).

	Normal	Mild	Moderate	*p*-Value
Operation time (minutes)	92.8 (92.1–93.5)	88.9 (86.3–91.5)	91.6 (86.9–96.4)	0.020
EBL (mL)	136.6 (131.3–141.8)	141.8 (123.3–160.2)	146.9 (117.6–176.2)	0.701
Transfusion rate (%)	14.0	27.0	32.5	< 0.001
Transfusion amount (mL)	648.1 (616.0–680.3)	1051.7 (412.4–1691.1)	2669.0 (1807.0–3531.0)	< 0.001
ICU admission (%)	0.7	0.4	6.0	0.002
Length of hospital stay (day)	15.1 (14.8–15.3)	16.7 (15.4–18.0)	17.4 (15.0–19.8)	0.008
Prolonged oozing (%)	9.6	10.9	2.8	0.219
30-day Readmission (%)	0.6	0.0	0.6	NA
1-year PJI (%)	1.6	1.2	3.3	0.431
1-year Mortality (%)	0.2	0.2	1.6	0.183

Values are presented as IPTW-adjusted weighted means (95% confidence intervals) for continuous variables and weighted percentages for categorical variables. Group comparisons were performed using IPTW-adjusted linear regression models for continuous variables and IPTW-adjusted logistic regression models for categorical variables. Overall group effects were evaluated using Wald tests. IPTW: inverse probability of treatment weighting, EBL: estimated blood loss, ICU: intensive care unit, PJI: periprosthetic joint infection, NA: not applicable.

**Table 3:** Multivariable logistic regression analysis in the primary study population.

PLT Group	RBC Transfusion		ICU Admission		1-Year PJI		1-Year Mortality	
	aOR (95% CI)	*p*-Value	aOR (95% CI)	*p*-Value	aOR (95% CI)	*p*-Value	aOR (95% CI)	*p*-Value
Normal	1 (reference)		1 (reference)		1 (reference)		1 (reference)	
Mild	1.48 (0.91–2.39)	0.108	0.52 (0.03–2.66)	0.535	0.95 (0.23–2.60)	0.925	1.54 (0.08–9.98)	0.703
Moderate	3.26 (1.51–6.59)	<0.001	5.46 (1.37–17.41)	0.008	3.14 (0.75–8.92)	0.061	3.06 (0.15–22.11)	0.343

Models were adjusted for clinically relevant covariates identified in univariable analyses. Normal platelet count (≥150,000/µL) was used as the reference group. aOR: adjusted odds ratio, CI: confidence interval, RBC: red blood cell, PLT: platelet count, ICU: intensive care unit, PJI: periprosthetic joint infection.

## Data Availability

The datasets used and/or analyzed during the current study are available from the corresponding author on reasonable request.

## Supplementary Materials

The following supporting information can be downloaded at: https://www.mdpi.com/article/10.3390/jcm15166187/s1, Table S1: Multivariable logistic regression analysis identifying independent predictors of RBC transfusion in the primary study population; Table S2: Multivariable logistic regression analysis identifying independent predictors of ICU admission in the primary study population; Table S3: Multivariable logistic regression analysis identifying independent predictors of 1-year PJI in the primary study population; Table S4: Multivariable logistic regression analysis identifying independent predictors of 1-year mortality in the primary study population; Table S5: Sensitivity analysis including the Severe group (IPTW adjusted); Table S6: Demographic data and outcomes of the Severe group (unadjusted).
