# Supplementary material for: Preoperative Thrombocytopenia Increases In-Hospital Burden but Not Postoperative Adverse Events After Total Knee Arthroplasty
Source: J Clin Med. 2026 Aug 10;15(16):6187. doi: 10.3390/jcm15166187 (PMC13512983; doi:10.3390/jcm15166187)
Supplement: Supplementary file 1 [file jcm-15-06187-s001.zip › jcm-4429338-supplementary.pdf]

Supplementary Table S1 Multivariable logistic regression analysis identifying independent predictors of RBC transfusion in the primary study population

	Univariable		Multivariable	
	OR (95% CI)	*P*-value	aOR (95% CI)	*P*-value
PLT group				
Normal	1 (reference)		1 (reference)	
Mild	2.08 (1.48-2.88)	<0.001	1.48 (0.91-2.39)	0.108
Moderate	5.18 (3.08-8.65)	<0.001	3.26 (1.51-6.59)	<0.001
Preoperative Hb	0.67 (0.63-0.71)	<0.001	0.59 (0.55-0.65)	<0.001
Age	1.01 (0.99-1.02)	0.302		
Sex				
Female	1 (reference)		1 (reference)	
Male	0.51 (0.37-0.69)	<0.001	1.25 (0.78-1.96)	0.353
Height	0.96 (0.95-0.98)	<0.001	0.99 (0.97-1.01)	0.320
Weight	0.97 (0.97-0.98)	<0.001	0.99 (0.98-1.00)	0.150
CCI Score	1.13 (0.99-1.30)	0.064		
AntiPLT/Anticoagulant	1.22 (1.00-1.49)	0.052		
Bilaterality				
Unilateral	1 (reference)		1 (reference)	
Bilateral	2.78 (2.29-3.39)	<0.001	5.46 (4.20-7.14)	<0.001
Anesthesia				
Spinal	1 (reference)			
General	0.86 (0.70-1.04)	0.127		
EBL	1.01 (1.01-1.02)	<0.001	1.01 (1.01-1.02)	<0.001
TXA use	0.08 (0.06-0.10)	<0.001	0.07 (0.05-0.09)	<0.001
Drain use	1.38 (0.95-2.09)	0.106		

RBC: red blood cell, PLT: platelet count, Hb: hemoglobin, CCI: Charlson Comorbidity Index, TXA: tranexamic acid, EBL: estimated blood loss, OR: odds ratio, aOR: adjusted odds ratio, CI: confidence interval.

Supplementary Table S2 Multivariable logistic regression analysis identifying independent predictors of ICU admission in the primary study population

		Univariable		Multivariable	
		OR (95% CI)	*P*-value	aOR (95% CI)	*P*-value
PLT group					
	Normal	1 (reference)		1 (reference)	
	Mild	0.76 (0.04-3.65)	0.787	0.52 (0.03-2.66)	0.535
	Moderate	10.6 (3.03-29.11)	<0.001	5.46 (1.37-17.41)	0.008
Preoperative Hb		0.72 (0.59-0.91)	0.003	0.82 (0.65-1.04)	0.094
Age		1.01 (0.95-1.07)	0.770		
Sex					
	Female	1 (reference)			
	Male	2.16 (0.84-4.95)	0.083		
Height		0.95 (0.90-1.01)	0.090		
Weight		1.00 (0.97-1.04)	0.794		
CCI Score		1.06 (0.61-1.85)	0.835		
AntiPLT/Anticoagulant		4.12 (1.89-9.44)	<0.001	2.68 (1.15-6.46)	0.024
Bilaterality					
	Unilateral	1 (reference)			
	Bilateral	0.59 (0.25-1.31)	0.208		
Anesthesia					
	Spinal	1 (reference)			
	General	1.43 (0.64-3.10)	0.373		
EBL		1.00 (1.00-1.00)	0.046	1.00 (1.00-1.00)	0.016
TXA use		0.13 (0.05-0.28)	<0.001	0.15 (0.06-0.34)	<0.001
Drain use		0.95 (0.28-5.95)	0.947		

ICU: intensive care unit, PLT: platelet count, Hb: hemoglobin, CCI: Charlson Comorbidity Index, TXA: tranexamic acid, EBL: estimated blood loss, OR: odds ratio, aOR: adjusted odds ratio, CI: confidence interval.

Supplementary Table S3 Multivariable logistic regression analysis identifying independent predictors of 1-year PJI in the primary study population

	Univariable		Multivariable	
	OR (95% CI)	*P*-value	aOR (95% CI)	*P*-value
PLT group				
Normal	1 (reference)		1 (reference)	
Mild	0.96 (0.23-2.63)	0.941	0.95 (0.23-2.60)	0.925
Moderate	3.26 (0.78-9.23)	0.052	3.14 (0.75-8.92)	0.061
Preoperative Hb	1.00 (0.85-1.20)	0.958		
Age	1.02 (0.98-1.07)	0.270		
Sex				
Female	1 (reference)			
Male	1.12 (0.51-2.19)	0.761		
Height	1.00 (0.97-1.04)	0.775		
Weight	1.02 (1.00-1.05)	0.089		
CCI Score	1.10 (0.75-1.61)	0.627		
AntiPLT/Anticoagulant	1.79 (1.04-3.05)	0.033	1.78 (1.02-3.03)	0.037
Bilaterality				
Unilateral	1 (reference)			
Bilateral	1.40 (0.83-2.41)	0.210		
Anesthesia				
Spinal	1 (reference)			
General	0.92 (0.51-1.59)	0.764		
EBL	1.00 (1.00-1.00)	0.817		
TXA use	0.90 (0.49-1.80)	0.759		
Drain use	1.41 (0.52-5.81)	0.566		

PJI: periprosthetic joint infection, PLT: platelet count, Hb: hemoglobin, CCI: Charlson Comorbidity Index, TXA: tranexamic acid, EBL: estimated blood loss, OR: odds ratio, aOR: adjusted odds ratio, CI: confidence interval.

Supplementary Table S4 Multivariable logistic regression analysis identifying independent predictors of 1-year mortality in the primary study population

		Univariable		Multivariable	
		OR (95% CI)	*P*-value	aOR (95% CI)	*P*-value
PLT group					
	Normal	1 (reference)		1 (reference)	
	Mild	2.28 (0.12-12.9)	0.441	1.54 (0.08-9.98)	0.703
	Moderate	7.62 (0.40-43.72)	0.059	3.06 (0.15-22.11)	0.343
Preoperative Hb		0.68 (0.49-0.99)	0.033	0.78 (0.54-1.15)	0.198
Age		1.08 (0.97-1.20)	0.167		
Sex					
	Female	1 (reference)			
	Male	2.92 (0.62-11.12)	0.130		
Height		1.03 (0.94-1.11)	0.555		
Weight		0.94 (0.89-1.02)	0.212		
CCI Score		1.72 (0.67-4.49)	0.265		
AntiPLT/Anticoagulant		3.19 (0.84-12.9)	0.084		
Bilaterality					
	Unilateral	1 (reference)			
	Bilateral	0.32 (0.05-1.33)	0.157		
Anesthesia					
	Spinal	1 (reference)			
	General	0.24 (0.01-1.32)	0.181		
EBL		1.00 (0.99-1.00)	0.940		
TXA use		0.12 (0.03-0.47)	0.003	0.15 (0.03-0.57)	0.007
Drain use		NA	NA		

PLT: platelet count, Hb: hemoglobin, CCI: Charlson Comorbidity Index, TXA: tranexamic acid, EBL: estimated blood loss, OR: odds ratio, aOR: adjusted odds ratio, CI: confidence interval, NA: not applicable.

Supplementary Table S5 Sensitivity analysis including the Severe group (IPTW adjusted)

	Normal	Mild	Moderate + Severe	*P*-value
Operation time (minutes)	92.8 (92.1-93.5)	89.3 (86.4-92.2)	91.4 (86.9-95.9)	0.068
EBL (ml)	136.7 (131.5-142.0)	143.0 (123.0-163.1)	148.3 (120.3-176.3)	0.676
Transfusion rate (%)	14.0	28.2	36.8	<0.001
Transfusion amount (ml)	648.8 (616.4-681.1)	976.9 (465.3-1,488.6)	2,982.7 (2,171.0-3,794.4)	<0.001
ICU admission (%)	0.7	0.3	6.4	<0.001
Length of hospital stay (day)	15.1 (14.8-15.3)	16.8 (15.6-17.9)	18.8 (16.1-21.6)	<0.001
Prolonged oozing (%)	9.5	12.3	7.1	0.449
30-day Readmission (%)	0.6	0.0	0.5	NA
1-year PJI (%)	1.6	1.3	3.0	0.578
1-year Mortality (%)	0.2	0.2	1.4	0.207

Sensitivity analysis performed by combining patients with severe thrombocytopenia (<50,000/μL) with the Moderate group.

Values are presented as IPTW-adjusted weighted means (95% confidence intervals) for continuous variables and weighted percentages for categorical variables. Group comparisons were performed using IPTW-adjusted linear regression models for continuous variables and IPTW-adjusted logistic regression models for categorical variables. Overall group effects were evaluated using Wald tests.

IPTW: inverse probability of treatment weighting, EBL: estimated blood loss, ICU: intensive care unit, PJI: periprosthetic joint infection, NA: not applicable.

Supplementary Table S6 Demographic data and outcomes of the Severe group (unadjusted)

Baseline characteristics		
Number		6
Age (year)		72.5 (64;76)
Sex		
	Male	1 (16.7%)
	Female	5 (83.3%)
Height (cm)		150.2 (146.8;162.1)
Weight (kg)		57.5 (53.5;62.2)
Baseline lab		
	PLT ($\times 10^3/\mu L$)	40 (28;43)
	Hb (g/dL)	12.4 (11.0;12.7)
CCI score		3.0 (2.0;3.0)
AntiPLT/Anticoagulant		0 (0.0%)
TXA use		3 (50.0%)
Drain use		6 (100.0%)
Bilaterality		
	Unilateral	4 (66.7%)
	Bilateral	2 (33.3%)
Anesthesia		
	Spinal	3 (50.0%)
	General	3 (50.0%)

Outcomes	
Operation time (minutes)	104 (85;137)
EBL (ml)	175 (150;300)
Transfusion rate (%)	66.7%
Transfusion amount	4,160 (2,880;8,000)
ICU admission (%)	0.0%
Length of hospital stay (day)	29 (16;45)
Prolonged oozing (%)	33.3%
30-day Readmission (%)	0.0%
1-year PJI (%)	0.0%
1-year Mortality (%)	0.0%

Values are presented as median (interquartile range) for continuous variables and number (%) for categorical variables.

PLT: platelet count, Hb: hemoglobin, CCI: Charlson Comorbidity Index, TXA: tranexamic acid, EBL: estimated blood loss, ICU: intensive care unit, PJI: periprosthetic joint infection.
